# Recent Advances in the Emission and Functions of Plant Vegetative Volatiles

**DOI:** 10.3390/molecules21020124

**Published:** 2016-01-22

**Authors:** Fang Dong, Xiumin Fu, Naoharu Watanabe, Xinguo Su, Ziyin Yang

**Affiliations:** 1 Guangdong Food and Drug Vocational College, Longdongbei Road 321, Tianhe District, Guangzhou 510520, China; dongfangxyz@163.com; 2 Key Laboratory of South China Agricultural Plant Molecular Analysis and Genetic Improvement & Guangdong Provincial Key Laboratory of Applied Botany, South China Botanical Garden, Chinese Academy of Sciences, Xingke Road 723, Tianhe District, Guangzhou 510650, China; fuxiumin@scib.ac.cn; 3 Graduate School of Science and Technology, Shizuoka University, 3-5-1 Johoku, Naka-ku, Hamamatsu 432-8561, Japan; watanabe.naoharu@shizuoka.ac.jp

**Keywords:** biosynthesis, ecological function, emission, multiple stress, plant volatiles, signaling

## Abstract

Plants synthesize and emit a large variety of volatile organic compounds, which possess extremely important ecological functions. In most case, most plant volatiles are liquids, rather than gases, at room temperature. Some volatiles are emitted “on demand” when plants, especially vegetative parts, are exposed to abiotic or biotic stress. In this review, we summarize some of the highlights of plant vegetative volatile emission and functions research published during the past few years.

## 1. Introduction

Plants synthesize and emit a large variety of volatile organic compounds. In principle, plant volatiles are low molecular weight metabolites (<300 Da) with a relatively low boiling point (<260 °C). These volatiles can be emitted from flowers, leaves, fruits, and roots into the atmosphere or soil, allowing the plant to interact with other organisms [1]. Until now, more than 1700 volatiles have been identified from more than 90 plant families, which contain approximately 1% of all plant specialized metabolites currently known [2]. According to the chemical structure, plant volatiles can be classed into hydrocarbons, alcohols, aldehydes, ketones, ethers, and esters. In the research field of plant science, plant volatiles are generally divided into volatile terpenes, volatile phenylpropanoid/benzenoid, and volatile fatty acid derivatives according to the different biosynthetic pathways. The synthesis of plant volatiles involves the removal of hydrophilic moieties and oxidation/hydroxylation, reduction, methylation, and acylation reactions [3]. The current researches on plant volatiles are mostly concentrated on answering the following questions, (1) how are plant volatiles formed? (2) how are plant volatiles emitted? (3) why do plants emit volatiles? or what are functions of plant volatiles? There are many reports and reviews [1,2,3,4,5,6,7,8] on the three issues above. In general, the answers can be briefly summarized as (1) plant volatiles are formed from the mevalonate/2-C-methyl-d-erythritol 4-phosphate pathway, shikimate pathway, and lipoxygenase pathway; (2) endogenous circadian clock and environmental factors such as biotic stresses and abiotic stresses can induce the emission of plant volatiles; (3) The emitted plant volatiles possess diverse ecological functions including attraction of pollinators, direct defense against herbivores, attraction of natural enemies of herbivores, and within-plant or plant-plant signaling. The rapid progress in technologies for metabolomics, proteomics, transcriptomics, and genomics allows the formation of plant volatiles to be rigorously investigated at the biochemical and molecular levels. Researchers are paying more attention to elucidating the emission and functions of plant volatiles, especially vegetative volatiles.

## 2. Volatile Emission from Vegetative Parts

### 2.1. Herbivore Induced Events Relating to Volatile Emission from Vegetative Parts

Plant volatiles emitted from vegetative parts are known to be involved in many types of biotic interactions. When herbivores attack plants, plants are subjected to mechanical damage and herbivore derived elicitors, which can induce a series of events in plants. Several herbivore derived elicitors of induced plant volatile emission have been identified, including fatty acid-amino acid conjugates [9,10], β-glucosidase [11], inceptin [12], caeliferins [13], and an unidentified heat-labile constituent [14]. These reported elicitors are identified in chewing herbivores, whereas piercing-sucking herbivore derived elicitors of induced plant volatile emission are not reported yet. This may be due to the fact that it is not easy to determine the amount of elicitors from the small size of piercing-sucking herbivores. So far, no direct evidence indicates that piercing-sucking herbivores contain the elicitors inducing plant volatile emission, but β-glucosidase was tentatively identified in the rice brown planthopper, and proposed as a potential elicitor inducing emission of rice volatiles [15]. In plants, the glycosidically-bound volatiles occur in vacuoles, whereas beta-glycosidases were observed to be localized in cell walls and the cavity areas among cells [16]. This compartmentation of substrates and enzymes in plant cells leads to the rare available evidences of hydrolysis of glycosidically-bound volatiles in intact plants [17]. Therefore, an exogenous β-glucosidase from herbivores possibly meets glycosidically-bound volatiles in plants through the piercing-sucking action of herbivores.

**Figure 1:**
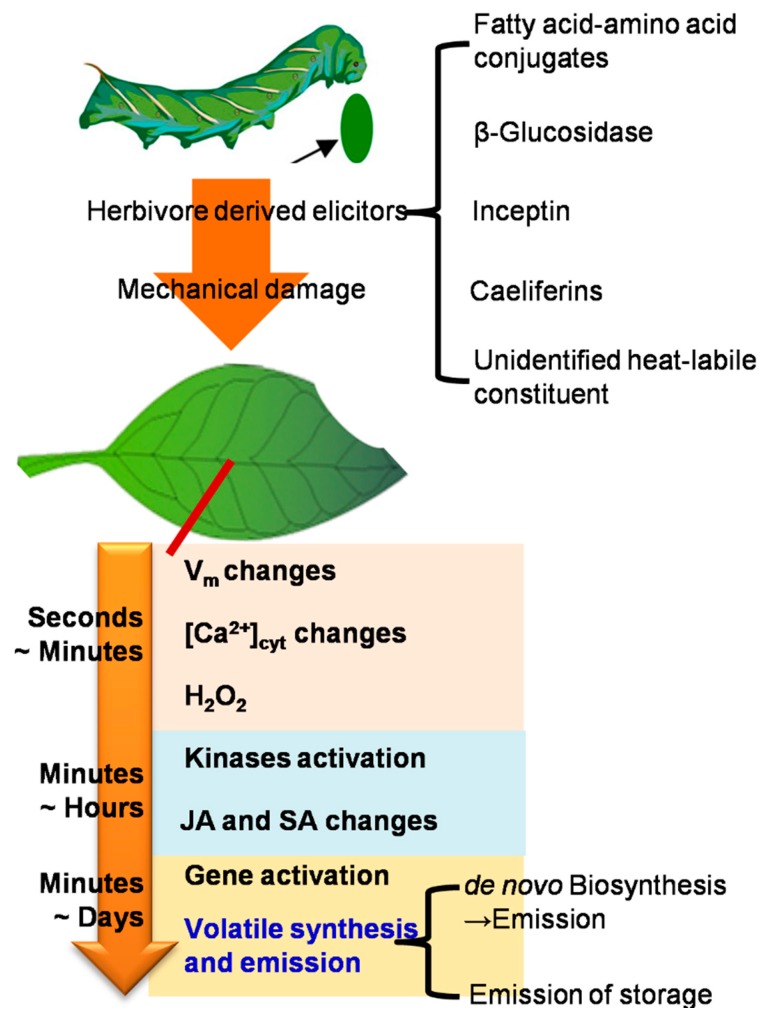
The events during the period from herbivore attack to volatile emission (The figure was drawn based on the reference [18]). JA, jasmonic acid. SA, salicylic acid.

The events during the period from herbivore attack to volatile emission (Figure 1) represent generally the first occasions from which measurements can be made. Maffei *et al.* (2007) well summarized the earliest events within the first seconds to minutes, which are responsible for recognition and triggering of signal transduction pathways of the plant–insect interaction [18]. The earliest events measurable are plasma transmembrane potential, immediately followed by changes in the intracellular cytosolic Ca^2+^ concentration and the formation of H_2_O_2_, which take place within seconds or minutes after attack and are mediated by insect oral secretions. Although Ca^2+^ influx has been demonstrated in plant–insect interactions, the role of anion and proton channels is not yet clear. Herbivore attack induces activation of the NADPH oxidase complex that generates the anion superoxide, which is quickly converted to H_2_O_2_ under the action of superoxide dismutase. H_2_O_2_ can accumulate in the extracellular matrix or enter the cell, afterwards induce a variety of late events such as kinases activation, and phytohormones jasmonic acid (JA) and salicylic acid (SA) signaling, which are detectable within minutes or hours. Kinases mediate subsequent steps in the interaction between plants and insects and are connected to later steps of phytohormone signaling. In plants, mitogen-activated protein kinases (MAPKs) regulate cellular responses to both external and endogenous stimuli in eukaryotes, and play an important role in the signaling of biotic stresses, pathogens and plant hormones. In addition, calcium-dependent protein kinasesare regularly involved in signal transduction of a variety of biotic and abiotic stresses. These kinases subsequently enhance transcript levels of genes involved in JA, SA, JA-Ile, and ethylene biosynthesis, which in turn enhance levels of these phytohormones [19]. The genes involved in formation of plant volatiles can be activated by the phytohormones, especially JA, which can lead to the high emission of herbivore-induced volatiles [20].

### 2.2. Multiple Stresses Induced Volatile Emission

Individual biotic and abiotic stresses, such as high temperature, high light, and herbivore attack, are well known to increase the emission of volatiles from vegetative parts of plants. In nature, plants are exposed to multiple stresses, either simultaneously or sequentially, causing much more complex volatile profiles than have usually been investigated in the individual stress-induced volatiles. However, in current researches, stresses have usually been considered as single or independent factors. Much less is known about the effect of multiple or co-occurring stress factors. When two or more stresses co-occur, in some cases, their effects are additive, while in other cases the influence of one stress may have priority [21]. A study on maize indicated the additive effects of biotic and abiotic stresses. In maize, the combination of high temperature and simulated herbivore infection resulted in higher volatile emission than individual stress [22]. Besides co-effects of biotic and abiotic stresses, different biotic stresses such as different herbivore attackers can affect the emission of herbivore-induced volatiles and, consequently, the attraction of carnivorous natural enemies of the inducing herbivores [23]. For instance, using a Y-Tube olfactometer, it was found that predatory mirid bugs and predatory mites prefer the herbivore-induced volatiles blends emitted by pepper plants and lima bean plants infested by two herbivore species (aphids and spider mites, caterpillars and spider mites) over the blends emitted by plants infested by either herbivore species separately [23], which may be due to the antagonism or synergia between JA and SA signaling pathways. Such crosstalk between phytohormone pathways also occurs when plants are subject to simultaneous attack by both herbivores and pathogens [24]. For example, in maize, fungal infection reduced the emission of volatiles induced by herbivore alone by about 50%, possibly suggesting a diversion of plant resources from anti-herbivore to presumptive anti-pathogen defenses. The authors hypothesized that fungal infection could stimulate the SA based signal transduction pathway which would reduce signaling through the herbivore-triggered JA pathway because of negative crosstalk [24]. These observations provide some evidences that multiple attackers can enhance, attenuate, or otherwise alter stress-induced volatile responses. However, more studies are needed to understand whether volatiles emission induced by multiple stresses is due to interlinkages among phytohormones such as JA, SA, and ethylene.

### 2.3. Effects of Green Leaf Herbivore Attack on Floral Scent Emission

Emissions of vegetative parts induced by green leaf herbivore attack have attracted much attention of researchers. Some researchers have directed their interest to investigate whether green leaf herbivore attack can affect emissions of floral parts. Effmert *et al.* (2008 [25]) reported *Nicotiana* *suaveolens* plants retained the quality of the volatile organic compounds’ composition as well as the quantity and emission patterns of their floral volatile organic compounds when the green leaf tissues experienced considerable damage by *Manduca* *sexta*. Moreover, there was no immediate floral response or delayed interference with the floral volatile organic compounds’ production/emission due to green leaf herbivory [25]. The authors proposed that metabolism in flowers at and post-anthesis is an autonomous process and is independent of metabolic changes in green leaves in *Nicotiana* *suaveolens* plants. By this sustaining mechanism, *Nicotiana* *suaveolens* plants ensure sexual reproduction even under unfavorable conditions [25]. However, not all plants have such similar phenomena. Theis *et al.* (2009) reported that mechanical simulation of chewing increased volatile terpenoid emission from male flowers [26]. Kessler *et al.* (2010) found that damage by *Manduca* spp. caterpillars caused reduced emission of the floral volatile benzyl acetone along with major changes in flower phenology [27]. These reports suggest that vegetative feeding by chewing herbivores can result in floral volatile emissions that are either enhanced, reduced, or unaffected. Furthermore, recent finding demonstrates that phloem-feeding herbivory can affect floral volatile emission, and that the outcome of interaction between herbivory and floral chemistry may differ depending on the herbivore’s feeding mode and degree of specialization [28].

## 3. Function of Volatiles from Vegetative Parts

### 3.1. Vegetative Volatiles Reduce Negative Effects of Stress on Plants

Several positive functions have been described for volatiles in vegetative plant tissues.

The first one is resistance to high temperatures and oxidative stress. Volatiles are known to be involved in many types of biotic interactions, but they, especially volatile isoprenoids, also play important but relatively unappreciated roles in abiotic stress responses [29]. High temperature is known to reduce leaf photosynthetic rate in many tree species. In contrast to non-emitting birch leaves, isoprene-emitting aspen leaves are more resistant against heat stress, which may be due to the fact that isoprene production increases tolerance of high temperatures [30]. Similarly, in exposure to oxidative stress, non-emitting transgenic tobacco plants showed a classical ozone-induced cell death response, whereas isoprene-emitting plants resisted better against ozone-induced damage [30]. Based on these observations, Vickers *et al.* proposed a plausible mechanism, *i.e.*, the “single biochemical mechanism for multiple stressors”, which is that abiotic stress responses generally involve production of reactive oxygen species in plant cells, and volatile isoprenoids mitigate the effects of oxidative stress by mediating the oxidative status of the plant, and the protective effect against abiotic stress is exerted through direct or indirect improvement in resistance to damage by reactive oxygen species [29]. A recent investigation on influences of heavy metal stress on plant volatiles also supported the “single biochemical mechanism for multiple stressors” model, and suggested that heavy metal stress is a prime factor for herbivore-induced plant volatile emission, and Cu stress correlated with increased levels of reactive oxygen species in roots and priming of herbivore-induced JA in leaves [31].

The second function is direct defense against herbivores. Many reports indicate that volatiles emitted from vegetative tissue can act as direct repellents of herbivores. For instance, green leaf volatiles can improve plant resistance against herbivores and fungal pathogen [32], and blends of monoterpenes, sesquiterpenes or green leaf volatiles were able to deter ovipositing females of several lepidopteran species [21]. Monoterpenoids, especially geraniol, can induce apoptosis-like cell death [33,34], which is caused as a defense reaction against bacterial infection [35].

The third function is attraction of herbivore enemies. Plants can defend themselves against herbivores by attracting natural enemies of the herbivores. This can be proofed by the evidences that transgenic plants engineered to produce specific terpenes and green leaf volatiles are involved in enemy attraction [32,36]. Moreover, in some case, a single gene can be sufficient to mediate the indirect defense of plants against herbivore attack, namely attraction of herbivore enemies. For instance, transcripts of *tps10*, a terpene synthase forming herbivory-induced sesquiterpene hydrocarbon, were found to be restricted to herbivore-damaged maize. Overexpression of *tps10* in *Arabidopsis thaliana* resulted in plants emitting high quantities of sesquiterpene products. The parasitic wasps preferred transgenic *Arabidopsis* that emits TPS10 sesquiterpenes in contrast to wild type after interaction with the plants [32].

The fourth function is within-plant or plant to plant signaling. Airborne communication among neighboring plants has been a controversial topic for many years, but there are now multiple examples in the literature where plants responded to signals mediated by volatiles emitted from neighboring plants under herbivore attack [37]. The controversy surrounding the plant-to-plant interactions also stimulated another research regarding intra-plant signaling among different organs. Since the studies of Narváez-Vásquez and Ryan (2004) showing how wounding triggers an increase in defenses in distant leaves, herbivore-induced within-plant signaling has usually been assumed to be transmitted via vascular connection [38]. Heil and Bueno (2007) demonstrated that herbivore-induced volatiles can serve as an external signal for within-plant (Lima bean) signaling [39]. Also, Frost and coworkers (2007) showed that within-plant (hybrid poplar) signaling mediated by volatiles can overcome vascular constraints to systemic signaling, and suggested that intra-plant signaling may have equal or greater ecological significance than signaling between plants [40]. These reports provide evidence that volatiles involved in signaling can improve defense abilities of neighboring tissues or plants and, thus, reduce the damage of neighboring tissues or plants. Although it is unclear how volatiles improve defense abilities of neighboring tissues or plants, there are a few reports investigating effects of volatiles on neighboring tissues or plants at gene or metabolite levels. Yao *et al.* (2011) found that UV-C-irradiated plants produce a volatile signal, such as methyl salicylate, or methyl jasmonate, or one unidentified volatile compound, which trigger an increase in genome instability in neighboring nonirradiated *Arabidopsis thaliana* plants. This volatile signal is interspecific, as UV-C-irradiated *Arabidopsis* plants transmit genome destabilization to naive tobacco (*Nicotiana* *tabacum*) plants and *vice versa* [41]. Dong *et al.* (2011) preformed a principal component analysis of metabolites (*m*/*z* 70–1000) in undamaged tea (*Camellia sinensis*) leaves exposed or not exposed to herbivore-induced volatiles, and found that external signaling via herbivore-induced volatiles may lead to more drastic changes in the metabolite spectrum of tea leaves than internal signaling via vascular connections [42]. Quite recently, Sugimoto *et al.* (2014) obtained a breakthrough finding on how plants receive volatiles and, consequently, how they fortify their defenses [43]. Undamaged tomato plants absorbed the airborne (*Z*)-3-hexenol emitted from neighboring conspecific plants exposed to herbivore attack and subsequently converted the alcohol to (*Z*)-3-hexenylvicianoside. The glycoside negatively affected the performance of common cutworms and suppressed their growth and survival rates [43]. The accumulation of glycoside in the receiver plants explained the defense acquired via “smelling” their neighbors. In addition, the authors found that (*Z*)-3-hexenylvicianoside biosynthesis was independent of JA signaling, revealing a previously unidentified mechanism of plant defense [43].

### 3.2. Quality or Quantity of Volatiles and Plant Defense Against Stress

**Figure 2:**
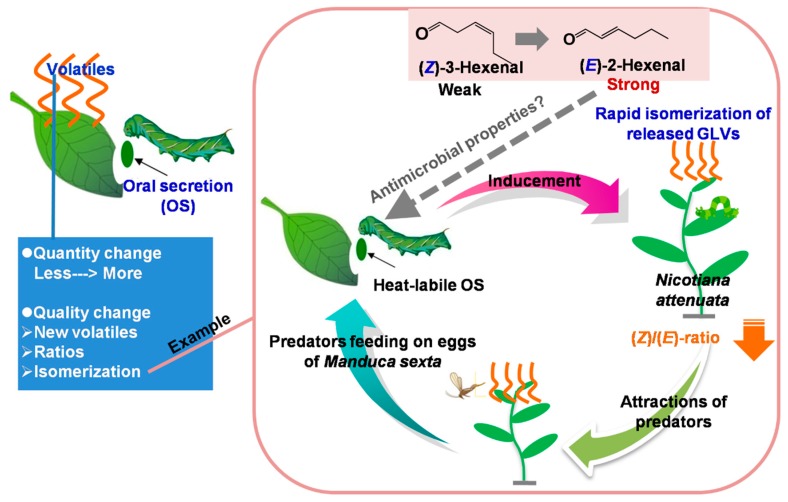
Emission patterns of volatiles induced by herbivore attack and an example of the relationship between a change in quality of herbivore-induced volatiles and plant defense (The figure was drawn based on the reference [14]).

Herbivore induced plant volatiles have several different metabolic origins, of which the isoprene-derived terpenoids and fatty acid-derived green leaf volatiles are the best-studied classes [2]. Terpenoids are released with a delay from the whole plant, not just attacked leaves, after a few hours or with the plant’s next photosynthetic phase. Because of their delay, the terpenoids likely function in the long distance attraction of carnivores. Unlike terpenoids, green leaf volatiles were immediately released from wounded leaves. Therefore, green leaf volatiles likely provide rapid information about the exact location of a feeding herbivore [44]. In general, herbivore attack induces an increase in plant volatile emission. Less is known about the effects of the quality of volatiles on plant defense against herbivore attack. Bruce *et al.* (2010 [45]) reported an unusual case that an African forage grass can release (*Z*)-3-hexenyl acetate as a major volatile, and, surprisingly, after stemborer ovipositing, the major released volatile (*Z*)-3-hexenyl acetate reduced, whereas other minor volatiles did not show significant changes. In the test of behavioral responses of parasitoids, parasitoids prefer volatiles from grass with oviposition than healthy grass. In addition, in oviposition bioassays, grass with eggs was less preferred for subsequent oviposition by stemborer than grass without eggs. These results suggest that changes in volatile ratios can alter the ability of herbivores to locate their host [45]. Moreover, Allmann *et al.* (2010) reported that attack by the specialist herbivore *Manduca* *larvae* and the addition of their oral secretions to mechanical wounds induce a rapid (*Z*)/(*E*) isomeric change in the green leaf volatiles release of *Nicotiana* plants. This change lowers the (*Z*)/(*E*) ratio of the green leaf volatiles blend, and increases the predation rate of the predator that is feeding on eggs of the herbivore [14] (Figure 2). On the other hand, the larvae may benefit from the enhanced antimicrobial properties of a green leaf volatiles blend enhanced in (*E*)-2-hexenal [46] (Figure 2). These findings suggest that, in some cases, insect responses are dependent on the quality of volatile emission rather than merely the quantity in this multitrophic interaction.

## 4. Concluding Remarks and Perspectives

**Figure 3:**
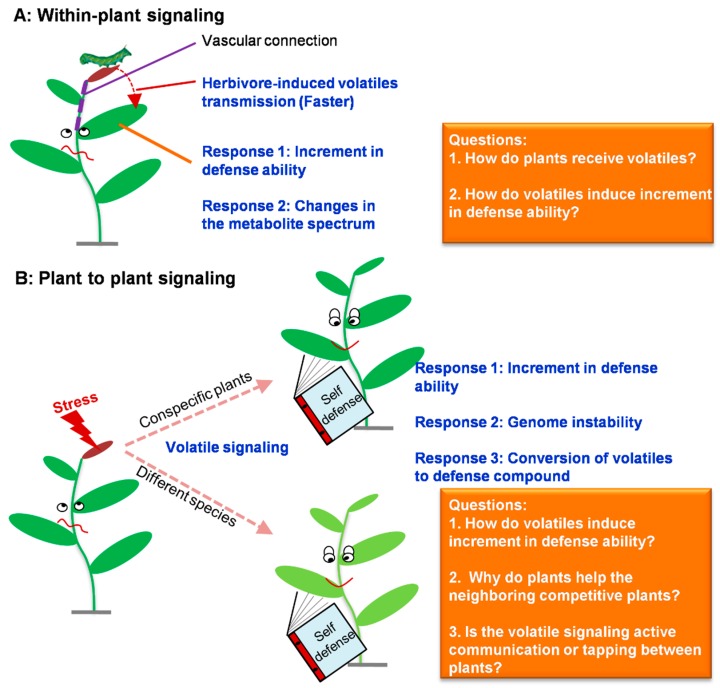
Summary on involvement of volatiles in within-plant or plant to plant signaling (The figure was drawn based on references [37,38,39,40,41,42,43]) and the derived questions.

This paper summarizes some of the highlights of plant vegetative volatile emission and functions research published during the past few years. The profile of the events from herbivore attack to plant vegetative volatile emission is basically characterized. However, to date, how volatiles are released from plant cells is largely unknown. Before being emitted into the environment, plant volatiles must cross the membrane, the aqueous cell wall, and, sometimes, the cuticle. Volatiles are primarily nonpolar compounds which preferentially partition into membranes, making diffusion into aqueous compartments slow, although it is presumed that volatiles move through each barrier via passive diffusion. However, Widhalm *et al.* (2015) proposed that volatiles must cross multiple cellular compartments to reach the environment, and biological mechanisms involved in trafficking other hydrophobic compounds must contribute to volatile emission, which may reduce barrier resistances [47]. Further studies on the impact of cuticle composition on volatile emission are required. On the issue of plant vegetative volatile functions, several important questions on within-plant or plant to plant signaling should be addressed in future studies (Figure 3): (1) How do plants receive volatiles? (2) How do volatiles induce increment in defense ability? (3) Why do plants help the neighboring competitive plants? (4) Is the volatile signaling active communication or tapping between plants?
